# Symptom severity, neighborhood crime, and neural correlates of reappraisal in patients with major depression and social anxiety

**DOI:** 10.1017/S0033291726104474

**Published:** 2026-05-15

**Authors:** Cope Feurer, Jagan Jimmy, Melissa Uribe, Katie L. Burkhouse, Stewart A. Shankman, K. Luan Phan, Olusola Ajilore, Heide Klumpp

**Affiliations:** 1 UNC: The University of North Carolina at Chapel Hill, USA; 2 The Ohio State University, USA; 3 Arizona State University, USA; 4 Penn State University: The Pennsylvania State University, USA; 5 Northwestern University, USA; 6 University of Illinois Chicago, USA

**Keywords:** fMRI, major depression, neighborhood adversity, reappraisal, social anxiety, symptoms

## Abstract

**Background:**

Cognitive reappraisal deficits are a transdiagnostic risk factor for major depressive disorder (MDD) and social anxiety disorder (SAD) and are observed in patients with these disorders at the neural level. Preliminary research suggests less activation of prefrontal regions during reappraisal (vs. viewing) of negative stimuli associates with overall symptom severity in patients with MDD or SAD, however, this is not reliably observed across studies. Consistent with research showing that reappraisal may only be adaptive when employed to cope with uncontrollable adversity, this study sought to examine whether neighborhood-level adversity (i.e. socioeconomic disadvantage, crime) moderated the relation between internalizing symptom severity and neural correlates of reappraisal.

**Methods:**

This study included patients with a current diagnosis of MDD (*n* = 51) or SAD (*n* = 39). Patients completed measures of symptom severity as well as an emotion regulation task while in the scanner to assess neural activation during reappraisal. Patients’ addresses were geocoded to assess neighborhood socioeconomic disadvantage and crime.

**Results:**

Results indicated that greater symptom severity was associated with decreased activation of key prefrontal regions underlying reappraisal, but only for patients living in neighborhoods characterized by high levels of personal (i.e. violent) crime. Unexpectedly, the opposite was found for patients living in low-crime neighborhoods, such that greater symptom severity was associated with increased neural activation during reappraisal (vs. viewing) of negative stimuli.

**Conclusions:**

Findings highlight the critical importance of considering patients’ neighborhood contexts when evaluating associations between symptom severity and neural correlates of reappraisal in patients with internalizing disorders.

## Introduction

Major depressive disorder (MDD) and social anxiety disorder (SAD) are highly impairing and prevalent psychiatric disorders, with lifetime prevalence of 18% and 13%, respectively (Kessler et al., 2012). There is significant variability in symptom severity within these internalizing disorders, and higher symptom severity is associated with greater functional impairment (Fried & Nesse, 2014; McKnight et al., 2016) and lower quality of life (Gao, Su, Sweet, & Calabrese, 2019; Lochner et al., 2003). Therefore, understanding factors that contribute to variance in symptom severity across MDD and SAD is critical for informing more effective therapeutic interventions.

Patterns of neural activation during cognitive reappraisal may provide important insights into these underlying mechanisms. Cognitive reappraisal, the process by which an individual decreases their emotional response to a stimulus by modifying their thoughts (Gross, 2015), is an adaptive regulation strategy associated with lower depression and social anxiety symptoms in community samples (Aldao & Nolen-Hoeksema, 2010), and deficits in cognitive reappraisal are observed in patients with MDD and SAD (Dryman & Heimberg, 2018; but see Arditte Hall, Quinn, Vanderlind, & Joormann, 2019). Furthermore, patients with internalizing disorders show altered neural responses when reappraising (contrasted with viewing) negative stimuli, such that they often exhibit decreased activation of key neural regions supporting reappraisal (e.g. dorsolateral prefrontal cortex [dlPFC], ventrolateral prefrontal cortex [vlPFC], dorsal anterior cingulate cortex [dACC]) compared to healthy controls (Blair et al., 2012; De la Peña-Arteaga et al., 2021; Goldin et al., 2009; Keller et al., 2022; Ziv et al., 2013). However, the extent to which these group differences are observed varies, suggesting the presence of heterogeneity that may relate to transdiagnostic symptom severity in patients with MDD or SAD.

Neuroimaging work provides some evidence that individual differences in brain activity when reappraising, versus viewing, negative images relate to internalizing symptom severity, though findings are inconsistent. One study using a whole-brain regression approach to examine relations with overall internalizing symptom severity (i.e. composite scores comprising both depression and anxiety symptoms) found that greater symptom severity was associated with less dACC activation during reappraisal of negative stimuli in a transdiagnostic sample of patients with MDD, SAD, or generalized anxiety disorder (Fitzgerald, Klumpp, Langenecker, & Phan, 2019). Similarly, another study using a regions of analysis (ROI) approach also found that less dACC activity during reappraisal correlated with greater internalizing symptoms in patients with depressive disorders (Edmiston et al., 2024). Another study did not test associations with dACC, but did find that depression and anxiety symptoms correlated with less vlPFC activation during reappraisal of negative stimuli in patients with MDD and posttraumatic stress disorder (Keller et al., 2022). However, a contrasting study did not find any associations between depression or anxiety symptoms and vlPFC response during reappraisal in MDD patients (De la Peña-Arteaga et al., 2021). Finally, a study of patients with SAD failed to detect any associations between social anxiety symptom severity and neural response during reappraisal at the whole-brain level (Goldin et al., 2009). Together, these findings suggest that although less prefrontal (i.e. dACC and vlPFC) activation during reappraisal versus viewing of negative images may correlate with internalizing symptom severity in some patients with either MDD or SAD, there may be patient subgroups for whom neural correlates of reappraisal do not relate to symptom severity, indicating the presence of moderating factors that may modulate this relation.

One potentially important moderating factor is a person’s environment. Research increasingly suggests that reappraisal is not universally adaptive, and that particular environmental contexts may play a role in individual differences in reappraisal facility. Specifically, forms of emotion-focused regulation, including reappraisal, are hypothesized to be an adaptive response to uncontrollable adversity (i.e. situations where environmental context cannot be altered), but not to controllable adversity (i.e. situations where one can alter their environment to reduce stress), where problem-solving strategies are more effective (Cheng, 2001; Troy et al., 2023). For example, emotion-focused regulation may be an adaptive response to a family member being diagnosed with cancer (i.e. uncontrollable adversity), whereas problem-solving may be a more adaptive response to a hostile work environment, if the person had the resources and ability to search for a better job. Supporting this, one study found that both depression and social anxiety symptoms were associated with reappraisal-situation match, such that participants with lower symptoms were more likely to use reappraisal in response to uncontrollable adversity (Haines et al., 2016). Other studies have similarly shown that greater reappraisal ability or use is associated with lower depression (Troy et al., 2017) and anxiety (Hittner, Rim, & Haase, 2019) symptoms in community samples, but only for participants with lower socioeconomic status (who often have less direct control over stressors in their lives; Kraus et al., 2012). Therefore, it is possible that lesser neural activity during reappraisal (vs. viewing) of negative stimuli may only be associated with symptom severity for patients with MDD or SAD who live in environmental contexts marked by high levels of uncontrollable adversity (i.e. for whom reappraisal can serve as an adaptive emotion regulation strategy).

One such environmental context that warrants attention is the adversity of the neighborhood. Although links with social anxiety remain largely unexplored, neighborhood-level adversity is linked with depression and anxiety symptoms and disorders (Baranyi et al., 2021; Barnett, Zhang, Johnston, & Cerin, 2018; Generaal et al., 2019; Richardson et al., 2015). Additionally, neighborhoods provide the overarching context in which individuals live their daily lives, but individuals are limited in their ability to control neighborhood-level adversities and may be limited in their choice of neighborhood due to individual-level socioeconomic factors (Kraus et al., 2012). Thus, neighborhood adversity is a salient and uncontrollable environmental context that may impact the extent to which neural correlates of reappraisal facility associate with symptom severity in MDD and SAD.

Some forms of neighborhood adversity may be particularly relevant when examining moderators of the relation between symptom severity and neural correlates of reappraisal. For example, both neighborhood socioeconomic disadvantage (Barnett et al., 2018; Generaal et al., 2019; Richardson et al., 2015) and neighborhood safety/ community violence (Baranyi et al., 2021; Barnett et al., 2018; Generaal et al., 2019) are linked with depression and anxiety risk. However, research also suggests that links between neighborhood socioeconomic disadvantage and internalizing risk may actually be driven by increased violent crime/safety concerns in these neighborhoods (Joshi et al., 2017; Stirling, Toumbourou, & Rowland, 2015). Although links between neighborhood adversity and neural activation during reappraisal remain unexplored, neighborhood socioeconomic disadvantage and violent crime have been shown to be associated with neural activation during emotion processing. For example, neighborhood socioeconomic disadvantage is associated with increased anterior cingulate cortex reactivity to fearful versus neutral faces in adults (Webb et al., 2023) and increased amygdala reactivity to threatening faces versus shapes in youth (Suarez et al., 2022). However, mirroring findings for internalizing symptoms, another study found that neighborhood violence mediated the relation between neighborhood disadvantage and amygdala reactivity to threatening faces versus shapes in youth (Suarez et al., 2024). Together, these findings highlight the importance of disentangling which specific forms of neighborhood adversity may moderate the relation between symptom severity and neural activation during reappraisal, and emphasize the potential unique role of neighborhood violent crime.

Therefore, the goal of this study was to examine whether neighborhood adversity moderates the relation between transdiagnostic internalizing symptom severity and neural activation during reappraisal of negative stimuli in patients with MDD or SAD. Based on evidence that reappraisal may only be associated with symptom severity in the context of uncontrollable adversity exposure, we hypothesized that greater symptom severity would be associated with less dACC and vlPFC activation during reappraisal of negative stimuli, but only for patients living in neighborhoods marked by high levels of adversity. Additionally, to determine which specific forms of neighborhood adversity may moderate this relation, we separately examined multiple forms of adversity as moderators (i.e. socioeconomic disadvantage, violent crime, nonviolent crime). Finally, we hypothesized that any observed associations between neural correlates of reappraisal and symptom severity would be transdiagnostic, such that they would be observed within both patient groups (i.e. MDD and SAD).

## Methods and materials

### Participants

Participants for this secondary analysis were 90 patients with MDD (*n* = 51) or SAD (*n* = 39) recruited as part of a larger study examining neural predictors and mechanisms of cognitive behavioral therapy treatment response between 2017 and 2022. Participants were required to be between the ages of 18 and 65, meet DSM-5 criteria for a current diagnosis of either SAD or MDD, and to exhibit elevated clinical symptoms (i.e. Liebowitz Social Anxiety Scale [LSAS; Liebowitz, 1987 score ≥60 for SAD patients; Hamilton Depression Rating Scale [HAMD; Hamilton, 1960 score ≥17; or Beck Depression Inventory-II [Beck, Steer, & Brown, 1996] score ≥16 for MDD patients). MDD clinical severity for the purposes of inclusion criteria was based on either the HAMD or BDI-II, given that these measures each capture different symptoms of depression (Möller, 2000). Diagnostic comorbidity was allowed, though SAD patients were not permitted to also have a current diagnosis of MDD, and vice versa. Exclusionary criteria included any history of psychosis, major medical illness, plan or intent for suicide or self-injurious behavior, any use of psychotropic medication in the 6 weeks prior to study entry, cognitive problems (e.g. traumatic brain injury, dementia), developmental disorders, or substance abuse or dependence in the moderate or severe range 6 months prior to study entry.

Participant average age was 28.12 (*SD* = 9.47) and 67.8% were female. Regarding race, participants identified as follows: 48.9% as White, 12.2% as Black, 14.4% as Asian, 1.1% as American Indian or Alaskan Native, 6.7% as more than one race, 13.3% as another racial identity, and 3.3% did not report their racial identity. Regarding ethnicity, 28.9% of participants identified as Hispanic/Latiné. Average years of education was 15.70 years (*SD* = 2.53). Demographic characteristics for participants with current MDD versus SAD are presented in Table 1. As MDD participants were older than SAD participants, age was included as a covariate in all analyses.

**Table 1.** Demographics and main study variables for participants with MDD and SAD

	MDD participants (*n* = 51)	SAD participants (*n* = 39)	*t*/χ^2^
Demographics			
Age	30.37 (10.56)	25.18 (6.90)	*t* = 2.67^**^
Sex			χ^2^ = 1.55
Female	70.6%	64.1%	
Male	29.4%	33.3%	
Not reported	0.0%	2.6%	
Ethnicity (Hispanic or Latiné)	31.4%	25.6%	χ^2^ = 0.35
Racial identity			χ^2^ = 6.69
White	52.9%	43.6%	
Black	7.8%	17.9%	
Asian	11.8%	17.9%	
Native American or Alaskan Native	0.0%	2.6%	
Multiracial/another identity	27.4%	17.9%	
Education (in years)	15.87 (2.61)	15.47 (2.44)	*t* = 0.74
Symptom measures			
LSAS	37.29 (17.57)	79.23 (16.27)	*t* = −11.58^***^
HAMD	14.16 (4.54)	8.64 (4.49)	*t* = 5.74^***^
Overall symptom severity	0.85 (0.22)	0.95 (0.22)	*t* = −2.14*
Neighborhood indices			
Area deprivation index	38.89 (18.66)	39.84 (21.03)	*t* = −0.23
Personal crime	149.80 (133.16)	203.46 (179.47)	*t* = −1.39
Property crime	133.76 (117.62)	132.18 (106.74)	*t* = 0.61

*Note*: SAD, social anxiety disorder; MDD, major depressive disorder. Overall symptom severity = summation of Liebowitz Social Anxiety Scale (LSAS) and Hamilton Depression Rating Scale (HAMD) proportion of maximum scaling (POMS) scores.

*p < .05; **p < .01; ***p < .001

### Procedures

Participants were recruited from a university-affiliated outpatient clinic and from the community using flyers and social media ads. Interested participants completed a phone screen and potentially eligible participants were invited to complete a baseline assessment to confirm study eligibility. At this assessment, informed consent was obtained and participants completed the Structured Clinical Interview for DSM-5 (First, Williams, Karg, & Spitzer, 2015) and symptom measures to confirm eligibility (which was confirmed by a Best-Estimate/Consensus Panel of at least three study staff members). Participants also completed the Emotion Regulation Task in the fMRI scanner to examine neural response during reappraisal of negative stimuli. All study procedures were approved by the University of Illinois at Chicago Institutional Review Board and complied with the Helsinki Declaration. Participants were compensated for their time.

### Symptom measures

Consistent with prior studies looking at transdiagnostic symptom severity in this sample (Feurer et al., 2021, 2024), an overall symptom severity score was created by summing clinician-assessed patient symptom scores (i.e. the HAMD and LSAS). Patient HAMD and LSAS scores were divided by the maximum observed score (i.e. proportion of maximum scaling [POMS] method; Little, 2013) to ensure they were on the same scale prior to summation.

### Neighborhood adversity

Patient neighborhoods were defined as the census block group corresponding to the address of their current residence at the time of study enrollment. Neighborhood socioeconomic disadvantage was indexed using the 2022 version of the Area Deprivation Index (ADI; Kind & Buckingham, 2018), which is a nationally normed factor score reflecting neighborhood socioeconomic disadvantage that is derived using 5-year data from the American Community Survey. Valid ADI data were not available for one patient, as ADI was unable to be calculated for their block group due to a high group quarters population as indicated by the 2022 ADI database. Therefore, analyses involving the ADI included a sample size of 89. Neighborhood crime was indexed using the 2023 CrimeRisk database (Applied Geographic Solutions, 2023), which is a nationally normed database created using 7 years of archival crime report data that reflects the risk of crime occurring in one’s neighborhood, with a score of 100 reflecting the national average. Based on meta-analytic work showing that neighborhood safety (i.e. violent crime) is linked with depression and anxiety (Baranyi et al., 2021), we examined personal (i.e. violent) and property (i.e. nonviolent) crime separately. Histograms showing frequency distributions for neighborhood Area Deprivation Index, personal crime, and property crime are presented in Supplementary Figure S1.

### Emotion regulation task

The Emotion Regulation Task (ERT) is a validated paradigm that probes neural mechanisms underlying reappraisal of standardized images of general negative content (Klumpp et al., 2017; Phan et al., 2005) and has been previously used to examine neural correlates of internalizing symptom severity (Fitzgerald et al., 2019). During this task, patients are presented with neutral or negative images from the International Affective Picture System (IAPS; Lang, Bradley, & Cuthbert, 1997) and are instructed to ‘Look Neutral’ or ‘Look Negative’ by naturally viewing neutral or negative images, respectively, without changing their emotional response, or ‘Reappraise Negative’ by decreasing their emotional response to negative images using situation-focused reappraisal to reinterpret the image. Consistent with prior research (Ochsner, Bunge, Gross, & Gabrieli, 2002; Phan et al., 2005), patients were trained in reappraisal strategies (i.e. think about the image in more positive terms or rationalize the content of the image) before completing the ERT using different IAPS images than those used during the scan. Instructions at the beginning of each task block lasted 5 seconds, followed by four images presented for 5 seconds each. After each block, participants rated ‘How negative do you feel?’ on a 5-point Likert scale. Behavioral performance on this task confirmed that participants in the current sample were successful in decreasing their negative affect in the ‘Reappraise Negative’ condition, compared to the ‘Look Negative’ condition (see Supplementary Material for analyses and results). Task blocks were interspersed with 20-second ‘baseline’ blocks comprising a fixation cross. The task consisted of 24 task blocks (8 per condition) presented in pseudorandom order across two runs.

### fMRI data collection and preprocessing

Scanning during the ERT was conducted on a 3.0 Tesla MR 750 scanner (General Electric Healthcare; Waukesha, WI) using a standard radiofrequency coil. See Supplementary Material for details on scanner parameters, preprocessing, and first-level modeling. The primary contrast of interest was Reappraise Negative > Look Negative.

### Analytic plan

An initial inspection of the data indicated that neighborhood personal and property crime were significantly skewed (*z* > 3.29). These variables were square-root and log-transformed, respectively, to satisfy assumptions of normality for statistical models.

Whole-brain regression analyses were conducted in SPM12 to examine the main and interactive effects of neighborhood adversity and symptom severity on neural response during reappraisal of negative stimuli. Separate models were conducted for each form of neighborhood adversity (i.e. ADI, personal crime, property crime). For each form of neighborhood adversity, we examined main and interaction effects in two separate models. In our ‘main effects’ model, we examined the main effects of composite symptom severity and neighborhood adversity on neural activation while including participant age as a covariate of no interest. Of note, results for the main effects of symptom severity and neighborhood adversity remain unchanged when looking at the main effects of symptoms and each form of neighborhood adversity in separate models, rather than controlling for their overlap. In our ‘interaction’ model, we also included the symptoms × neighborhood adversity interaction. Cluster size threshold for significance was determined via 3dClustSim (Cox, 1996) using a threshold of *α* < 0.05 and a voxel threshold of *p* < 0.001. Specifically, we used 3dClustSim (version 19.3.16) to estimate the cluster-size threshold using second-nearest neighbor clustering, two-sided thresholding, and the autocorrelation function (ACF), where the spatial ACF is estimated using 3dFWHMx. This voxel threshold (*p* < .001) was set to correct for multiple comparisons (i.e., Bonferroni correction = .05/6 = .008). A power analysis conducted in G*Power 3.1.9.7 indicated that the current sample was powered at 80% at a threshold of *p* < .001 to detect moderate (*f*^2^ = .20) interaction effects.

For significant clusters, we report on the number of voxels falling within regions as defined by the AAL 3 atlas. Mean cluster activation was extracted using MarsBaR (Brett, Anton, Valabregue, & Poline, 2002). Extracted clusters were submitted to the Statistical Package for Social Sciences (SPSS, version 29) to evaluate the direction and magnitude of significant activity and conduct simple slopes and regions of significance analyses to interpret significant interactions. Tests of sensitivity were also conducted in SPSS to examine whether findings were maintained when controlling for demographic characteristics (i.e., sex) and individual-level socioeconomic status (i.e., education level). To examine whether specific forms of neighborhood adversity uniquely interacted with symptom severity to predict cluster activation, further tests of sensitivity were conducted to examine whether findings were maintained when controlling for interactions between symptoms and each other form of neighborhood adversity. Additional follow-up analyses were conducted to clarify whether findings were driven by individual differences in neural activation during the Reappraise Negative or Look Negative conditions. Specifically, follow-up analyses in SPSS examined whether findings remained significant when looking at extracted cluster activation separately for the Reappraise Negative condition and the Look Negative condition. Finally, to examine whether observed findings were transdiagnostic, regression analyses in SPSS were conducted to examine whether findings for extracted clusters were observed when examining the MDD and SAD patient groups separately.

## Results

### Participant characteristics

Characteristics for participants with MDD and SAD are presented in Table 1. As seen, patients with MDD were older than patients with SAD, but there were no other demographic differences (i.e., sex, racial identity, ethnicity, education) between groups. As expected, MDD patients exhibited higher HAMD scores than SAD patients, whereas patients with SAD exhibited higher LSAS scores than patients with MDD. HAMD scores indicated that MDD patients exhibited depression symptoms in the mild range (Zimmerman et al., 2013). LSAS scores indicated that SAD patients exhibited social anxiety symptoms in the marked range (Liebowitz, 1987). Patient groups did not differ on neighborhood adversity.

### Preliminary analyses

Means, standard deviations, and correlations among main study variables are presented in Table 2. Untransformed means and standard deviations are presented for neighborhood crime to facilitate comparison with other studies.

**Table 2.** Correlations and descriptive statistics for main study variables

	1.	2.	3.	4.	5.	6.	7.	8.	9.	10.
1. Age	–									
2. Sex (% female)	.10	–								
3. Race/ethnicity (% non-Hispanic/Latiné White)	.14	.16	–							
4. Education (in years)	.18	.23*	.22*	–						
5. LSAS	−.33^***^	−.09	−.05	−.29^**^	–					
6. HAMD	.35	.10	.05	.01	−.42^***^	–				
7. Overall symptom severity	.03	.01	−.003	−.26*	.52^***^	.55^***^	–			
8. ADI	<.001	−.11	−.41^***^	−.22*	.15	.04	.19	–		
9. Personal crime	.02	−.22*	−.22*	.09	.06	.10	.15	.29^**^	–	
10. Property crime	.11	−.11	−.09	.33^**^	−.18	.20	.03	−.11	.69^***^	–
**Mean (*SD*)**	28.12 (9.47)	67.8%	41.1%	15.70 (2.53)	55.47 (26.89)	11.77 (5.27)	0.89 (0.22)	39.29 (19.59)	173.06 (156.30)	133.08 (112.42)
**Range**	18–60			12–24	0–114	1–24	0.33–1.36	4–90	4–774	9–840

*Note*: LSAS, Liebowitz Social Anxiety Scale; HAMD, Hamilton Depression Rating Scale. Overall symptom severity = summation of LSAS and HAMD proportion of maximum scaling (POMS) scores. ADI, Area Deprivation Index.

*p < .05; ^**^p < .01; ^***^p < .001.

### Moderator: area deprivation index

Results of the whole-brain regression analyses are presented in Table 3. None of the main or interactive effects of symptom severity or neighborhood ADI were significantly associated with neural activation during the ERT in the models examining neighborhood socioeconomic disadvantage.

**Table 3.** Results from whole-brain regression analysis

Area deprivation index						
			Peak coordinates			
	Anatomical regions	*k*	*x*	*y*	*z*	*z* score
Main effects model						
	*None*					
Symptoms × ADI model						
	*None*					

Personal crime						
			Peak coordinates			
	Anatomical regions	*k*	*x*	*y*	*z*	*z* score
Main effects model						
	*None*					
Symptoms × Crime model						
	Left precentral gyrus, postcentral gyrus, and inferior frontal gyrus (opercular part)	684	−56	6	22	4.71
	Left and right middle cingulate cortex/dorsal anterior cingulate cortex	372	12	−2	40	4.02
	Left and right medial superior frontal gyrus	288	−8	48	14	3.63

Property crime						
			Peak coordinates			
	Anatomical regions	*k*	*x*	*y*	*z*	*z score*
Main effects model						
	*None*					
Symptoms × Crime model						
	Left precentral gyrus and postcentral gyrus	252	−42	−12	36	3.95

*Note*: All models included patient age as a covariate of no interest.

### Moderator: personal crime

**Figure 1.**
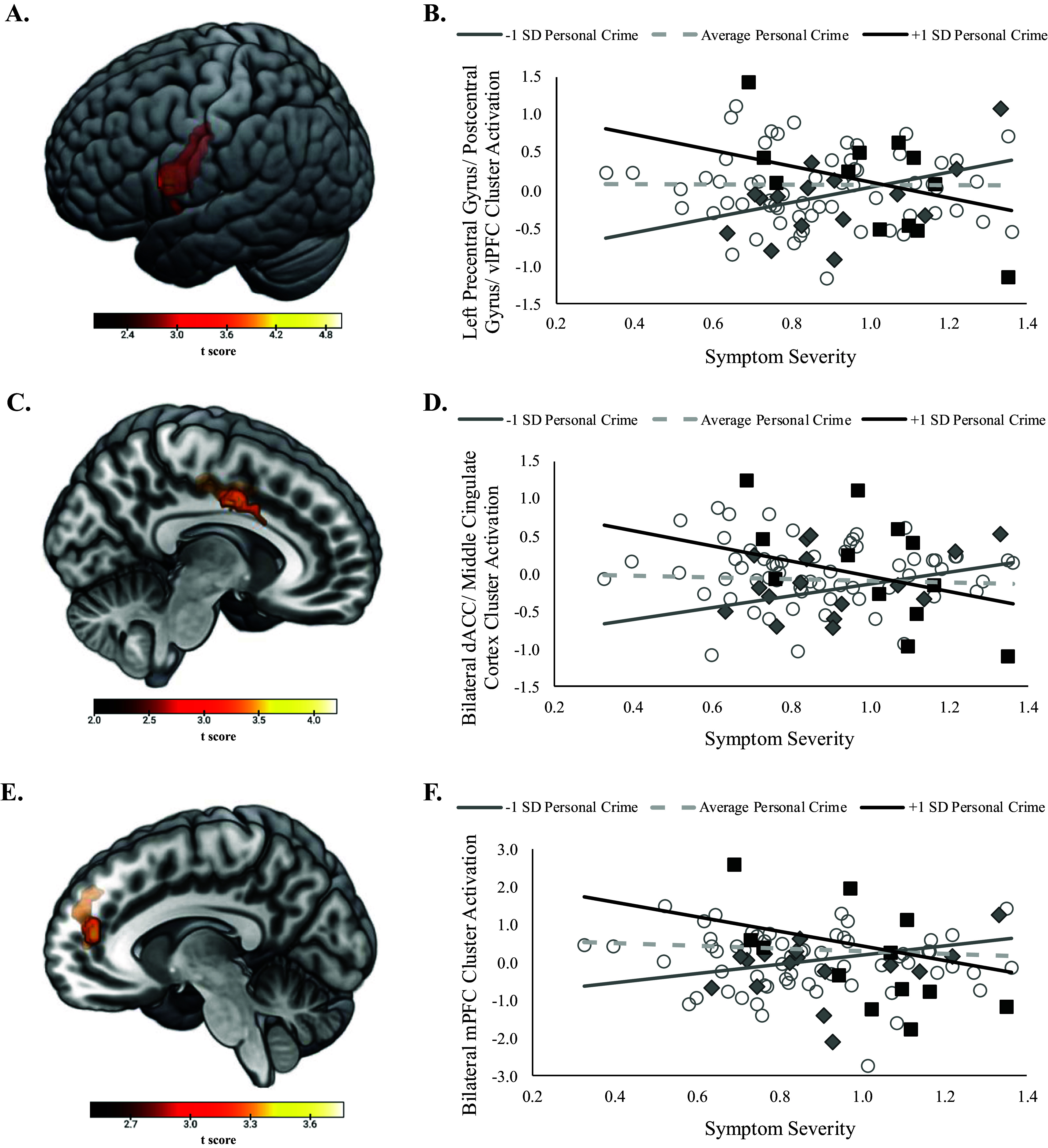
Significant activation of clusters comprised (a) left precentral and postcentral gyrus and ventrolateral prefrontal cortex (vlPFC), (c) bilateral dorsal anterior cingulate cortex (dACC) and middle cingulate cortex, and (e) bilateral medial prefrontal cortex (mPFC) from whole-brain regression analyses looking at the interaction between overall symptom severity and neighborhood personal crime controlling for patient age. Scatterplots and simple slopes looking at the relation between symptom severity and extracted cluster activation controlling for patient age for the (b) left precentral gyrus, (d) bilateral middle cingulate cortex, and (f) mPFC clusters. Simple slopes are presented at high (+1 *SD*), low (−1 *SD*), and average levels of neighborhood personal crime. Data points for scatterplots are categorized by ≥+1 *SD* (black squares), ≥−1 *SD* (grey diamonds), or <±1SD (white circles) for neighborhood personal crime for visualization purposes. Symptom severity = summation of LSAS and HAMD proportion of maximum scaling (POMS) scores.

Regarding personal crime models, the main effects of symptoms and neighborhood crime were not significantly associated with neural activation. However, the symptoms × neighborhood personal crime interaction significantly predicted activation of three clusters. The first cluster (peak [−56, 6, 22], *k* = 684 voxels, *z* = 4.71, *p* < .001) primarily comprised left precentral (*k* = 317 voxels) and postcentral gyrus (*k* = 214) and extending to left inferior frontal gyrus (i.e. vlPFC), opercular part (*k* = 100 voxels) (Figure 1a). Follow-up simple slope analyses (Figure 1b) indicated that symptom severity was negatively associated with cluster activation for participants living in neighborhoods with high (+1 *SD*) personal crime, *t*(85) = −3.39, *p* = .001, but positively associated with cluster activation for participants living in neighborhoods with low (−1 *SD*) personal crime, *t*(85) = 2.89, *p* = .005. Additionally, Johnson-Neyman regions of significance analyses (see Supplementary Figure S2a) indicated that symptoms were positively associated with cluster activation for patients living in neighborhoods with an untransformed CrimeRisk score <78.18 (28.9% of participants), but negatively associated with activation patients living in neighborhoods with an untransformed CrimeRisk score >206.78 (24.4% of participants). Follow-up tests of sensitivity indicated that simple slopes remained significant when statistically controlling for the influence of participant sex and education (highest *p* = .005).

The second cluster (peak [12, −2, 40], *k* = 372 voxels, *z* = 4.02, *p* < .001) comprised bilateral middle cingulate, encompassing both dACC (defined as AAL 3 middle cingulate anterior to *y* = 0; *k* = 107) and posterior middle cingulate (*k* = 265) (Figure 1c). Mirroring results from the first cluster, follow-up simple slope analyses (Figure 1d) revealed that greater symptom severity was associated with less dACC/ middle cingulate activation for participants living in high (+1 *SD*) personal crime neighborhoods, *t*(85) = −3.53, *p* < .001, but greater activation for participants living in low (−1 *SD*) personal crime neighborhoods, *t*(85) = 2.46, *p* = .02. Regions of significance analyses (see Supplementary Figure S2b) indicated that symptoms were positively associated with cluster activation for patients living in neighborhoods with an untransformed CrimeRisk score <60.83 (24.4% of participants), but negatively associated with activation patients living in neighborhoods with an untransformed CrimeRisk score >192.93 (27.8% of participants). Simple slopes remained significant in follow-up sensitivity analyses when independently statistically controlling for the influence of participant sex and education (highest *p* = .02).

The third cluster (peak [−8, 48, 14], *k* = 288 voxels, *z* = 3.63, *p* < .001) largely comprised bilateral medial superior frontal gyrus (i.e. medial prefrontal cortex [mPFC], *k* = 202) extending to left pregenual anterior cingulate cortex (*k* = 56) (see Figure 1e). Follow-up simple slopes (Figure 1f) revealed that symptom severity was negatively associated with mPFC activation for patients living in high (+1 *SD*) personal crime neighborhoods, *t*(85) = −3.68, *p* < .001, but positively associated with mPFC activation for patients living in low (−1 *SD*) personal crime neighborhoods, *t*(85) = 2.07, *p* = .04. Johnson–Neyman regions of significance analyses (see Supplementary Figure S2c) indicated that symptoms were positively associated with cluster activation for patients living in neighborhoods with an untransformed CrimeRisk score <44.18 (21.1% of participants), but negatively associated with activation patients living in neighborhoods with an untransformed CrimeRisk score >179.22 (32.2% of participants). Simple slopes remained significant when statistically controlling for participant sex and education in tests of sensitivity (highest *p* = .04).

#### Follow-up analyses controlling for interactions with neighborhood disadvantage and property crime

Results indicated that neighborhood personal crime uniquely interacted with symptom severity to significantly predict each extracted cluster activation when statistically controlling for interactions with either neighborhood disadvantage or property crime. See Supplementary Material for full details.

#### Follow-up analyses with individual betas for Reappraise Negative and Look Negative conditions

The symptoms × neighborhood personal crime interaction continued to significantly predict activation of the left precentral/postcentral gyrus/vlPFC cluster and of the mPFC cluster for the Reappraise Negative condition. Follow-up simple slopes showed that symptoms continued to be negatively associated with cluster activations for patients living in high-crime neighborhoods. For the Look Negative condition, the symptoms × neighborhood personal crime interaction only significantly predicted activation of the dACC/middle cingulate cluster, though simple slopes were not significant (lowest *p* = .11). See Supplementary Material for details.

#### Follow-up analyses within MDD and SAD patient groups

Follow-up analyses showed that the symptoms × neighborhood personal crime interaction significantly predicted activation of each extracted cluster when looking within the MDD and SAD patient subgroups separately. Follow-up simple slopes showed that symptoms continued to be negatively associated with cluster activations for patients living in high-crime neighborhoods across both patient groups and all extracted clusters. See Supplementary Material for details.

### Moderator: property crime

**Figure 2.**
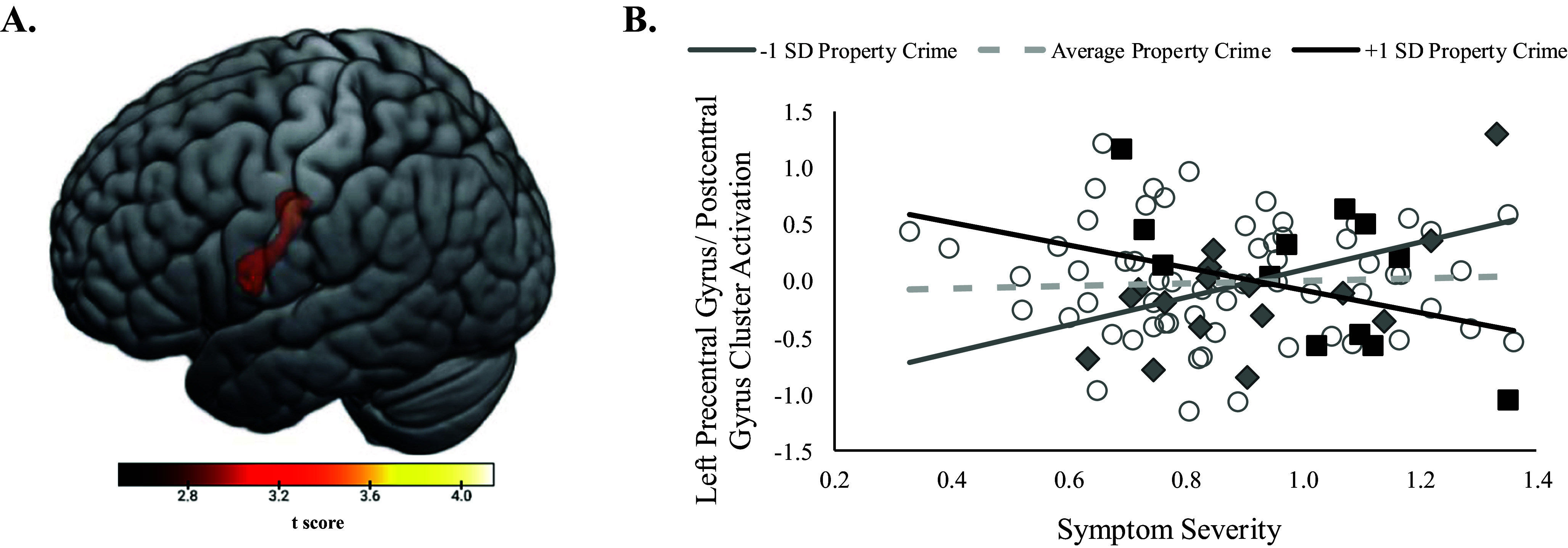
(a) Significant activation of a cluster comprised left precentral and postcentral gyrus from whole-brain regression analyses looking at the interaction between symptom severity and neighborhood property crime controlling for patient age. (b) Scatterplot and simple slopes looking at the relation between symptom severity and extracted left precentral/ postcentral gyrus cluster activation controlling for patient age. Simple slopes are presented at high (+1 *SD*), low (−1 *SD*), and average levels of neighborhood property crime. Data points for scatterplots are categorized by ≥+1 *SD* (black squares), ≥−1 *SD* (gray diamonds), or <±1SD (white circles) for neighborhood property crime for visualization purposes. Symptom severity = summation of LSAS and HAMD proportion of maximum scaling (POMS) scores.

In the models examining property crime, again, there was no main effect of participant symptom severity or neighborhood property crime on neural activation during the ERT. However, there was a significant symptoms × neighborhood property crime interaction that significantly predicted activation of a cluster similar to that observed for the personal crime model (peak [−42, −12, 36], *k* = 252 voxels, *z* = 3.95, *p* < .001) primarily comprised left postcentral (*k* = 184 voxels) and precentral gyrus (*k* = 56) (Figure 2a). Simple slopes analyses indicated that greater symptom severity was associated with less cluster activation for participants from high (+1 *SD*) crime neighborhoods, *t*(85) = −3.01, *p* = .004, but greater cluster activation for participants from low (−1 *SD*) crime neighborhoods, *t*(85) = 3.00, *p* = .004 (Figure 2b). Johnson–Neyman regions of significance analyses (see Supplementary Figure S3) indicated that symptoms were positively associated with cluster activation for patients living in neighborhoods with an untransformed CrimeRisk score < 74.75 (26.7% of participants), but negatively associated with activation patients living in neighborhoods with an untransformed CrimeRisk score > 157.69 (26.7% of participants). Findings remained significant when statistically controlling for covariates in follow-up tests of sensitivity (highest *p* = .007).

#### Follow-up analyses controlling for interactions with neighborhood disadvantage and personal crime

Results indicated that neighborhood property crime uniquely interacted with symptom severity to significantly predict cluster activation when statistically controlling for interactions with either neighborhood disadvantage or personal crime. However, simple slopes were no longer significant in the model controlling for personal crime. See Supplementary Material for details.

#### Follow-up analyses with individual betas for Reappraise Negative and Look Negative conditions

Follow-up analyses found that the symptoms × neighborhood property crime interaction significantly predicted extracted cluster activation during the Look Negative, but not the Reappraise Negative, condition. Simple slopes indicated that symptom severity was negatively associated with postcentral/precentral gyrus cluster activation during the Look Negative condition, but only for patients living in low-crime neighborhoods. See Supplementary Material for details.

#### Follow-up analyses within MDD and SAD patient groups

Follow-up analyses showed that the symptoms × neighborhood property crime interaction predicted cluster activation when looking within the SAD patient subgroup, but not the MDD subgroup. Simple slopes for SAD patients remained statistically significant for those living in both high- and low-crime neighborhoods. See Supplementary Material for details.

## Discussion

The goal of this secondary analysis was to examine whether neighborhood adversity moderated the relation between transdiagnostic symptom severity and neural activation during reappraisal of negative stimuli in patients with MDD or SAD. Results indicated that symptom severity was negatively associated with neural activation during reappraisal of (vs. viewing) negative stimuli, specifically for patients who lived in neighborhoods marked by high levels of crime. However, the opposite was true for patients living in low crime neighborhoods, such that symptom severity was positively associated with cluster activation. Tests of sensitivity indicated that findings remained significant when controlling for demographic factors and individual-level socioeconomic status, suggesting that findings were at least partially independent of these variables. Finally, results were replicated within both the MDD and the SAD subgroups, suggesting that neural activation during reappraisal of negative stimuli as a context-specific correlate of symptom severity may be transdiagnostic.

For patients living in high-crime neighborhoods, results revealed an association between greater symptom severity and lesser activation of multiple key brain regions previously shown to underlie cognitive reappraisal of negative stimuli (Messina, Bianco, Sambin, & Viviani, 2015). Specifically, greater symptoms were associated with less activation of clusters when reappraising versus viewing negative stimuli, including the vlPFC, dACC, and mPFC, which are involved in response selection of semantic information (Nee, Wager, & Jonides, 2007), conflict monitoring (Etkin, Egner, & Kalisch, 2011; Stevens, Hurley, & Taber, 2011), and emotional appraisal (Etkin et al., 2011), respectively. Symptom severity was also associated with lesser activation of the precentral and postcentral gyrus when reappraising versus viewing negative stimuli. These regions, involved in emotion perception (Kropf, Syan, Minuzzi, & Frey, 2019; Saarimäki et al., 2016), have been shown to exhibit altered activation during reappraisal when comparing patients with internalizing disorders to healthy controls (Picó-Pérez et al., 2017). Findings for vlPFC, dACC, and precentral gyrus mirror what has been observed in some prior research (Edmiston et al., 2024; Fitzgerald et al., 2019; Keller et al., 2022), and may clarify mixed findings regarding whether these regions reliably relate to overall symptom severity. Specifically, current findings highlight neighborhood adversity (i.e., crime) as an important context in which this association may be specifically observed.

Evidence that vlPFC, dACC, and mPFC consistently engage to support reappraisal in healthy samples (Messina et al., 2015), coupled with evidence that lower symptom severity was associated with greater cluster engagement during reappraisal in this study, suggests that greater activation of these key regions may be adaptive for patients living in an environment marked by uncontrollable adversity (i.e. high neighborhood crime). This is supported by a growing body of research showing that reappraisal deficits may predict symptom severity specifically in the context of uncontrollable adversity (Haines et al., 2016; Hittner et al., 2019; Troy et al., 2017) and suggests that patients with ‘intact’ neural mechanisms underlying reappraisal may be somewhat buffered against the deleterious impact of neighborhood crime. This said, neural correlates of reappraisal may reflect ability to effectively engage in this emotion regulation strategy when instructed, but not real-world frequency of use or reappraisal success, specifically in response to controllable versus uncontrollable stressors. Conclusions should remain tentative pending further exploration to better understand why lesser prefrontal activation during reappraisal relates to symptom severity in the context of neighborhood crime.

Unexpectedly, an opposite pattern of findings was observed for patients living in low crime neighborhoods, such that greater symptom severity was associated with greater activation of these clusters during reappraisal versus looking at negative stimuli. Although speculative, consistent with evidence that individuals with higher socioeconomic status have greater control over their environments, including their choice in neighborhoods (Kraus et al., 2012), it is possible that patients with the resources that allow them to live in low-crime neighborhoods also have greater control over other adversities in their lives. Therefore, reappraisal may be a less adaptive form of regulation for these individuals (Cheng, 2001; Troy et al., 2023). However, this study did not assess other forms of adversity or participant perceptions of controllability, therefore precluding the testing of this hypothesis. It is also important to note that simple slopes for the relation between symptom severity and cluster activation were not consistently significant for patients living in low-crime neighborhoods in follow-up sensitivity tests looking at neural activation during the Reappraise Negative and Look Negative conditions separately, therefore, these findings may be less robust. Future research is needed to further probe the nature of the relation between internalizing symptom severity and neural activation during reappraisal for MDD and SAD patients living in low crime neighborhoods.

The current study found that neighborhood crime, but not overall socioeconomic disadvantage, moderated the relation between symptom severity and neural activation during reappraisal of negative stimuli. Also, findings for neighborhood crime were largely driven by neighborhood personal (i.e. violent) crime rather than property crime. Although research has shown that both neighborhood socioeconomic disadvantage and crime are associated with depression and anxiety risk, neighborhood violent crime or safety concerns are more reliably linked with psychopathology risk than neighborhood socioeconomic disadvantage (Baranyi et al., 2021; Barnett et al., 2018; Generaal et al., 2019; Richardson et al., 2015). Therefore, it may be that neighborhood violent crime, specifically, may be an important neighborhood-level factor that may contextualize the relation between symptom severity and neural correlates of reappraisal in patients with MDD or SAD.

Findings should be interpreted with regard to some limitations. First, as noted earlier, this study did not assess for controllable forms of adversity, which may have helped to interpret the findings for patients living in low-crime neighborhoods. Second, patient neighborhood was defined as their current address at the time of study enrollment, however, it is unknown how long they had lived there or how much time they spend in their neighborhood. Third, although the current study extends prior work by showing that the relation between symptom severity and neural activation during reappraisal may be transdiagnostic and observed independently within patients with MDD and SAD, the sample sizes of these subgroups were relatively small, precluding testing of further moderation by diagnosis group at the whole-brain level. Studies with larger samples sizes are needed to examine three-way interactions between symptom severity, neighborhood adversity, and patient diagnosis group at the whole-brain level to examine if diagnosis-specific associations are also present. Fourth, given prior research linking community violence with externalizing behaviors (Fowler et al., 2009), it is important that future research examine whether findings also extend to externalizing symptoms. Fifth, although we interpreted higher symptom severity as maladaptive for participants living in high-crime neighborhoods, it is important to note that decreased social motivation and increased social avoidance, which are hallmark symptoms of depression and social anxiety, could be an adaptive response that promotes safety in high-threat environments. Future work should further disentangle internalizing symptoms from adaptive behavioral changes in the context of neighborhood crime. Finally, the current study is cross-sectional, precluding conclusions from being drawn regarding whether neural correlates of reappraisal prospectively predict the course of symptom severity in patients with MDD or SAD.

In conclusion, findings build from prior research on neural correlates of symptom severity in patients with MDD or SAD to help clarify mixed findings regarding the reliability of observed effects (De la Peña-Arteaga et al., 2021; Edmiston et al., 2024; Fitzgerald et al., 2019; Goldin et al., 2009; Keller et al., 2022). Specifically, findings suggest that patterns of neural engagement during reappraisal may be associated with either increased or decreased symptom severity in patients with MDD or SAD depending on the neighborhood context in which a patient is living. Furthermore, findings highlight neighborhood violent crime as a specific form of neighborhood adversity that may moderate this relation. If replicated, current findings may have important implications for the development of targeted and personalized treatment for patients with MDD or SAD to reduce symptom severity, based on environmental context. For example, to the extent that associations between symptoms and neural correlates of reappraisal differ by neighborhood context, neurostimulation interventions may aim to increase vlPFC and dACC activation for patients living in high violent crime neighborhoods, but decrease activation for patients in low-crime neighborhoods.

## Supporting information

10.1017/S0033291726104474.sm001 Feurer et al. supplementary material Feurer et al. supplementary material

## Supplementary material

The supplementary material for this article can be found at http://doi.org/10.1017/S0033291726104474.
