# Supplementary material for: Symptom severity, neighborhood crime, and neural correlates of reappraisal in patients with major depression and social anxiety
Source: Psychol Med. 2026 May 15;56:e157. doi: 10.1017/S0033291726104474 (PMC13200157; doi:10.1017/S0033291726104474)
Supplement: Feurer et al. supplementary material [file S0033291726104474sup001.docx]

**Supplemental Materials**

*Emotion Regulation Task Behavioral Performance*

A 2 (Group: MDD, SAD) × 3 (Condition: Look Neutral, Look Negative, Reappraise Negative) mixed ANOVA was conducted to confirm that participants were successful in their attempts to decrease their emotional responses using reappraisal and to examine whether participants in the MDD and SAD groups differed in their reappraisal ability. Results indicated that neither the main effect of Group, F(1, 87) = 1.74, *p* = .191, nor the Group × Condition interaction was significant, F(2, 174) = 1.47, *p* = .232. However, there was a main effect of Condition, F(2, 174) = 248.05, *p* < .001. Follow-up pairwise comparisons with a Bonferroni correction confirmed that reported negative emotion significantly differed across all conditions (all *p*s < .001) and participants were successful in their reappraisal. Specifically, participants showed the highest levels of negative emotion during the Look Negative condition (Mean = 2.93, SE = .086), followed by the Reappraise Negative condition (Mean = 2.42, SE = .082), and then the Look Neutral condition (Mean = 1.16, SE = .034).

*fMRI Data Collection and Preprocessing*

Blood oxygen-level dependent (BOLD) functional images were acquired using a gradient-echo echo-planar imaging sequence with the following parameters: repetition time (TR)=2 s, echo time (TE)=25 ms, flip angle=90⁰, field of view=22 × 22 cm, acquisition matrix 64 × 64; 44 axial, 3-mm-thick slices with no gap. The first 4 volumes from each run were discarded to allow for T1 equilibration effects. For anatomical localization, a high-resolution, T1-weighted volumetric anatomical scan was acquired.

Conventional preprocessing steps were used in Statistical Parametric Mapping (SPM12) software package (Wellcome Trust Centre for Neuroimaging, London www.fil.ion.ucl.ac.uk/spm). Images were temporally corrected to account for differences in slice time collection, spatially realigned to the first image of the first run, coregistered to the anatomical, normalized to a Montreal Neurological Institute (MNI) space using warping based on the anatomical, resampled to 2 × 2 × 2 mm voxels, and smoothed with an 8 mm isotropic Gaussian kernel. All participants were required to have no movement greater than 3-mm in any direction for analysis.

A general linear model identifying the Look Neutral, Look Negative, Reappraise Negative, and Fixation blocks was applied to the time series, convolved with the canonical hemodynamic response function and with a 128 s high-pass filter. First level models included 6 motion parameters as nuisance regressors.

*Follow-up Personal Crime Analyses Controlling for Interactions with Neighborhood Disadvantage and Property Crime*

Looking first at the extracted cluster comprised of left precentral and postcentral gyrus and vlPFC, the symptoms × neighborhood personal crime interaction continued to significantly predict cluster activation when controlling for either the symptoms × neighborhood disadvantage interaction, *t*(83) = -4.01, *p* < .001, or the symptoms × neighborhood property crime interaction, *t*(83) = -2.71, *p* = .008. Follow-up simple slopes for both models indicated that symptom severity continued to be negatively associated with cluster activation for patients living in high (+1 *SD*) crime neighborhoods (*ps* < .03) and positively associated with cluster activation for patients living in low (-1 *SD*) crime neighborhoods (*ps* < .03)*.*

Next, the symptoms × neighborhood personal crime interaction also continued to significantly predict activation of the extracted cluster (peak [12, -2, 40]) comprised of dACC/ middle cingulate when controlling for either the symptoms × neighborhood disadvantage interaction, *t*(83) = -4.05, *p* < .001, or the symptoms × neighborhood property crime interaction, *t*(83) = -3.33, *p* = .001. Follow-up simple slopes indicated that symptom severity continued to be negatively associated with cluster activation for patients living in high (+1 *SD*) crime neighborhoods (*ps* < .003) and positively associated with cluster activation for patients living in low (-1 *SD*) crime neighborhoods (*ps* < .02).

Finally, the symptoms × neighborhood personal crime interaction continued to predict activation for the extracted cluster comprised of mPFC when controlling for either the symptoms × neighborhood disadvantage interaction, *t*(83) = -3.85, *p* = .002, or the symptoms × neighborhood property crime interaction, *t*(83) = -3.06, *p* = .003. Follow-up simple slopes for both models indicated that symptom severity was negatively associated with cluster activation for patients living in high (+1 *SD*) crime neighborhoods (*ps* < .003). Symptom severity was positivity associated with cluster activation for patients living in low (-1 *SD*) crime neighborhoods when controlling for interactions with neighborhood disadvantage, *t*(83) = 2.03, *p* = .046, but not when controlling for interactions with property crime, *t*(83) = 1.87, *p* = .065.

*Follow-up Personal Crime Analyses with Individual Betas for the Reappraise Negative Condition*

Looking first at the extracted cluster comprised of left precentral and postcentral gyrus and vlPFC, the symptoms × neighborhood personal crime interaction predicted activation during the Reappraise Negative condition, *t*(85) = -2.73, *p* = .008. Follow-up simple slopes indicated that symptom severity was negatively associated with cluster activation for patients living in high (+1 *SD*) crime neighborhoods, *t*(85) = -2.91, *p* = .005, but was not associated with cluster activation for patients living in low (-1 *SD*) crime neighborhoods, *t*(85) = 1.16, *p* = .25.

Next, looking at the extracted cluster comprised of mPFC, the symptoms × neighborhood personal crime interaction also predicted activation during the Reappraise Negative condition, *t*(85) = -2.53, *p* = .01. Follow-up simple slopes indicated that symptom severity was negatively associated with cluster activation for patients living in high (+1 *SD*) crime neighborhoods, *t*(85) = -2.49, *p* = .01, but was not associated with cluster activation for patients living in low (-1 *SD*) crime neighborhoods, *t*(85) = 1.25, *p* = .21.

Finally, the symptoms × neighborhood personal crime interaction did not significantly predict activation of the extracted cluster (peak [12, -2, 40]) comprised of dACC/ middle cingulate during the Reappraise Negative condition, *t*(85) = -1.70, *p* = .09.

*Follow-up Personal Crime Analyses with Individual Betas for Look Negative Condition*

Analyses focusing on extracted cluster activation in the Look Negative condition indicated that the symptoms × neighborhood personal crime interaction did not significantly predict activation of the precentral/ postcentral gyrus/ vlPFC cluster, *t*(85) = 1.29, *p* = .20 or the mPFC cluster, *t*(85) = 0.43, *p* = .67. The symptoms × neighborhood personal crime interaction did significantly predict activation of the dACC/ middle cingulate cluster, *t*(85) = 2.02, *p* = .047. However, follow-up simple slopes indicated that symptom severity was not significantly associated with cluster activation for patients living in either high (+1 *SD*) crime, *t*(85) = 1.30, *p* = .20, or low (-1 *SD*) crime neighborhoods, *t*(85) = -1.61, *p* = .11.

*Follow-up Personal Crime Analyses: MDD Subsample*

Follow-up analyses were conducted to confirm whether findings were maintained when focusing on the subsample of patients with MDD. Analyses confirmed that the symptoms × neighborhood personal crime interaction significantly predicted activation of the precentral/ postcentral gyrus/ vlPFC cluster, *t*(46) = -2.64, *p* = .01, the mPFC cluster, *t*(46) = -2.74, *p* = .009, and the dACC/ middle cingulate cluster, *t*(46) = -2.84, *p* = .007. Simple slopes analyses for the precentral/ postcentral gyrus/ vlPFC cluster indicated that symptom severity was associated with cluster activation for patients living in high (+1 SD) crime neighborhoods, *t*(46) = -2.30, *p* = .03, but not low (-1 SD) crime neighborhoods, *t*(46) = 1.76, *p* = .08. Similarly, follow-up simple slopes for the mPFC cluster indicated that symptom severity was associated with cluster activation for patients living in high (+1 SD) crime neighborhoods, *t*(46) = -2.87, *p* = .006, but not low (-1 SD) crime neighborhoods, *t*(46) = 1.43, *p* = .16. Finally, simple slopes indicated that symptom severity was negatively associated with dACC/ middle cingulate cluster activation for patients living in high (+1 SD) crime neighborhoods, *t*(46) = -2.30, *p* = .03, but positively associated with cluster activation for patients living in low (-1 SD) crime neighborhoods, *t*(46) = 2.05, *p* = .046.

*Follow-up Personal Crime Analyses: SAD Subsample*

Looking next at the subsample of patients with SAD, mirroring the primary analyses, the symptoms × neighborhood personal crime interaction significantly predicted activation of the precentral/ postcentral gyrus/ vlPFC cluster, *t*(34) = -3.31, *p* = .002, the mPFC cluster, *t*(34) = -3.09, *p* = .004, and the dACC/ middle cingulate cluster, *t*(34) = -3.26, *p* = .003. Simple slopes analyses for the precentral/ postcentral gyrus/ vlPFC cluster indicated that symptom severity was associated with diminished cluster activation for patients living in high (+1 SD) crime neighborhoods, *t*(34) = -2.44, *p* = .02, but greater cluster activation for patients living in low (-1 SD) crime neighborhoods, *t*(34) = 2.25, *p* = .03. Follow-up simple slopes for the mPFC cluster indicated that symptom severity was associated with cluster activation for patients living in high (+1 SD) crime neighborhoods, *t*(34) = -2.52, *p* = .02, but not low (-1 SD) crime neighborhoods, *t*(34) = 1.89, *p* = .07. Similarly, simple slopes indicated that symptom severity was negatively associated with dACC/ middle cingulate cluster activation for patients living in high (+1 SD) crime neighborhoods, *t*(34) = -3.10, *p* = .004, but was not associated with cluster activation for patients living in low (-1 SD) crime neighborhoods, *t*(34) = 1.58, *p* = .12.

*Follow-up Property Crime Analyses Controlling for Interactions with Neighborhood Disadvantage and Personal Crime*

The symptoms × neighborhood property crime interaction continued to significantly predict postcentral/ precentral gyrus cluster activation when controlling for either the symptoms × neighborhood disadvantage interaction, *t*(83) = -4.01, *p* < .001, or the symptoms × neighborhood personal crime interaction, *t*(83) = -2.03, *p* = .046. Follow-up simple slopes indicated that when controlling for interactions with neighborhood disadvantage, symptom severity continued to be negatively associated with cluster activation for patients living in high (+1 *SD*) crime neighborhoods, *t*(83) = -2.86, *p* = .005, and positively associated with cluster activation for patients living in low (-1 *SD*) crime neighborhoods, *t*(83) = 3.24, *p* = .002. However, when controlling for interactions with personal crime, simple slopes were not significant for participants living in high (+1 *SD*) crime neighborhoods, *t*(83) = -1.71, *p* = .09, or low (-1 *SD*) crime neighborhoods, *t*(83) = 1.72, *p* = .09.

*Follow-up Property Crime Analyses with Individual Betas for Reappraise Negative and Look Negative Conditions*

Analyses indicated that the symptoms × neighborhood property crime interaction did not predict activation of the extracted postcentral/ precentral gyrus cluster for the Reappraise Negative condition, *t*(85) = -1.51, *p* = .14. However, the interaction was significant for the Look Negative condition, *t*(85) = 2.19, *p* = .03. Follow-up simple slopes indicated that symptom severity was negatively associated with cluster activation for patients living in low (-1 *SD*) crime neighborhoods, *t*(85) = -2.46, *p* = .02, but was not associated with cluster activation for patients living in high (+1 *SD*) crime neighborhoods, *t*(85) = 0.73, *p* = .47.

*Follow-up Property Crime Analyses: MDD and SAD Subsamples*

Looking first at the subsample of patients with MDD, results indicated that the symptoms × neighborhood property crime interaction did not predict activation of the extracted postcentral/ precentral gyrus cluster, *t*(47) = -1.72, *p* = .09. In contrast, the symptoms × neighborhood property crime interaction did significantly predict activation of the extracted postcentral/ precentral gyrus cluster for patients with SAD, *t*(35) = -3.16, *p* = .003. Follow-up simple slopes indicated that symptom severity was negatively associated with cluster activation for patients living in high (+1 *SD*) crime neighborhoods, *t*(35) = -2.38, *p* = .02, but was positively associated with cluster activation for patients living in low (-1 *SD*) crime neighborhoods, *t*(35) = 2.44, *p* = .02.

**
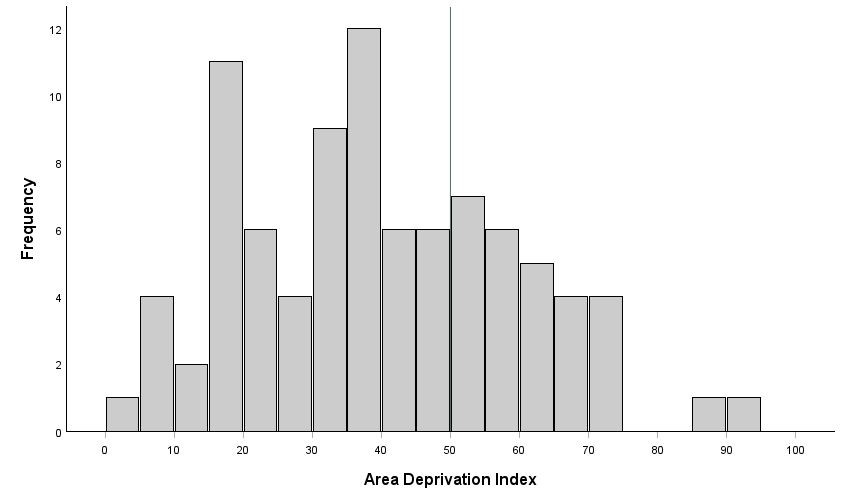
A.**

**
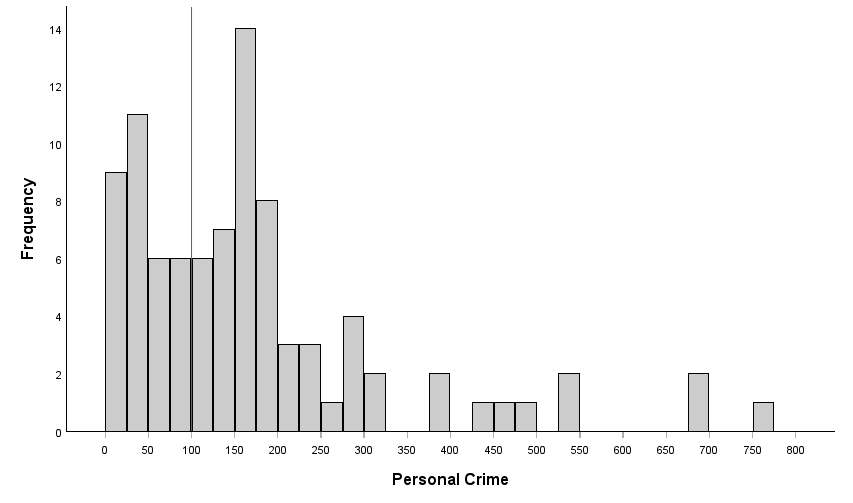
B.**

**C.**

**
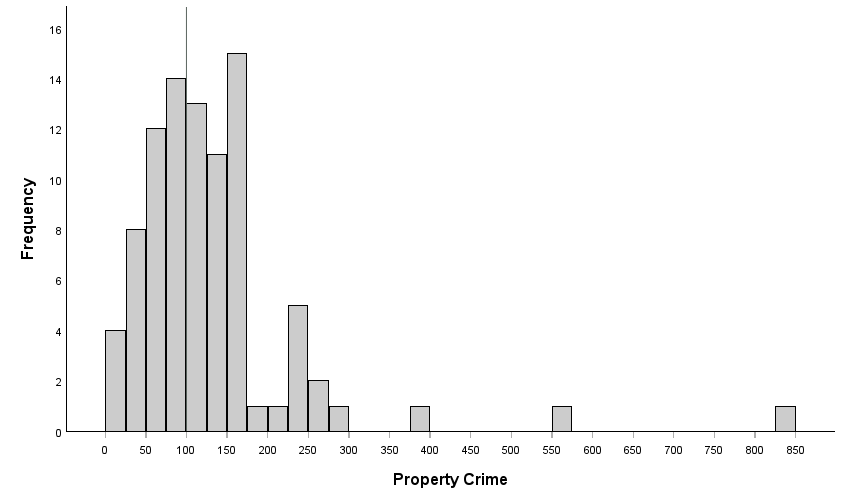
**

**Supplementary Figure 1.** Histograms depicting the frequency distribution of neighborhood (A) Area Deprivation Index, (B) Personal Crime, and (C) Property Crime. The vertical grey line denotes the national average.

**
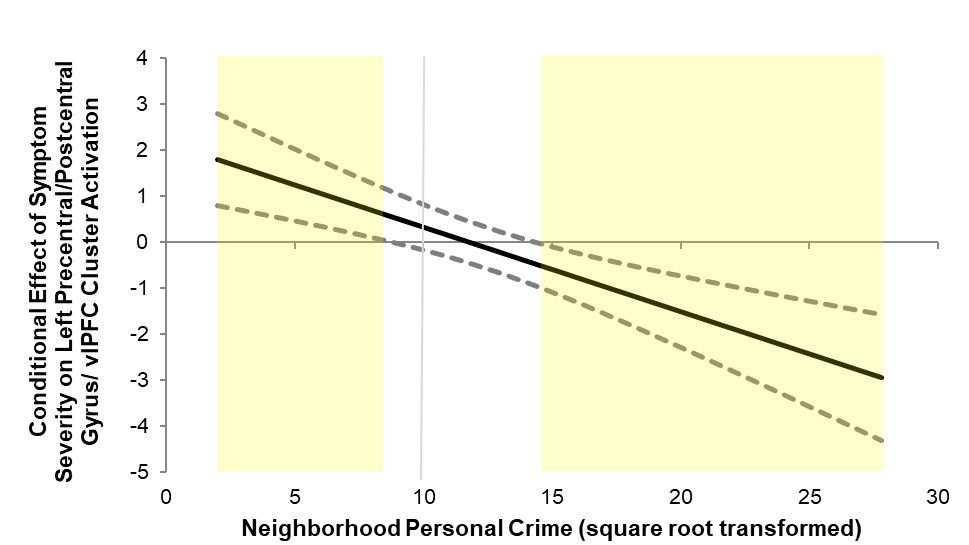
A.**

**
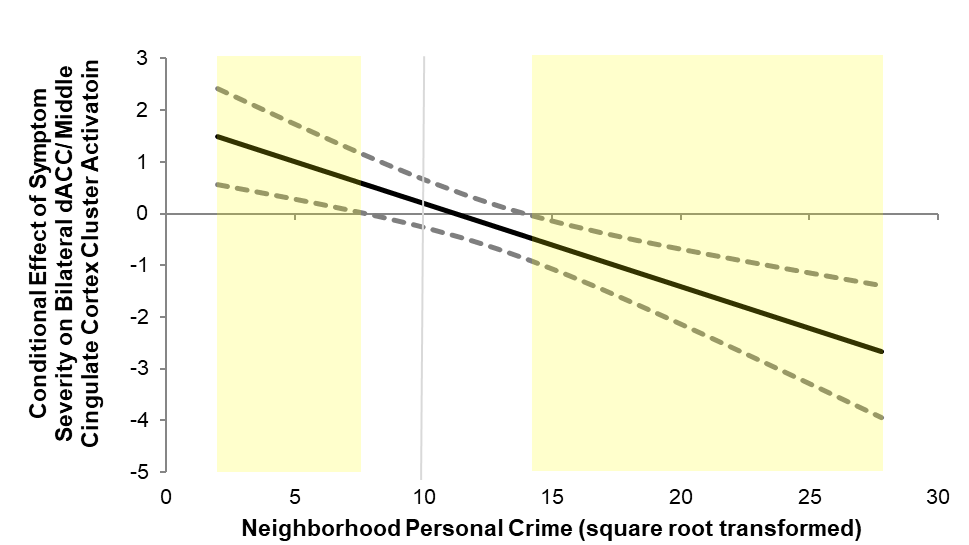
B.**

**
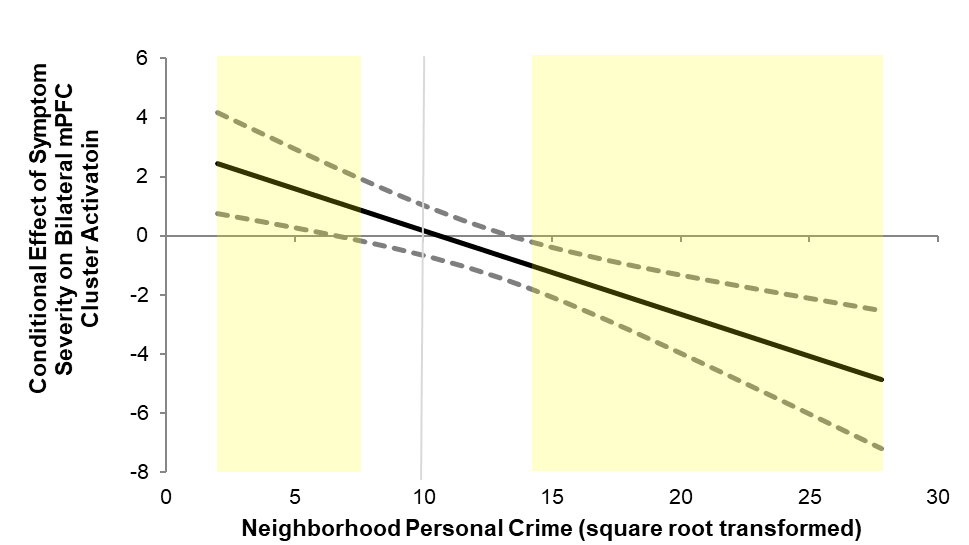
**

**C**.

**Supplementary Figure 2.** Johnson-Neyman plots showing the conditional effect of symptom severity on cluster activation at different levels of neighborhood personal crime for (A) left precentral and postcentral gyrus and ventrolateral prefrontal cortex (vlPFC), (B) bilateral dorsal anterior cingulate cortex (dACC) and middle cingulate cortex, and (C) bilateral medial prefrontal cortex (mPFC). Regions of significance are highlighted. The vertical grey line denotes the national average for neighborhood crime.


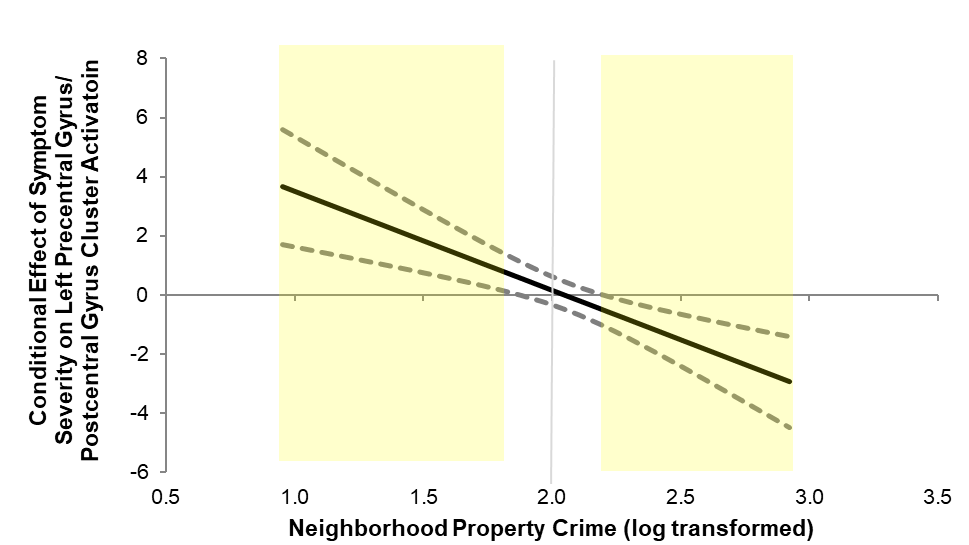


**Supplementary Figure 3.** Johnson-Neyman plot showing the conditional effect of symptom severity on left precentral and postcentral gyrus cluster activation at different levels of neighborhood property crime. Regions of significance are highlighted. The vertical grey line denotes the national average for neighborhood property crime.
